# Detection of Electroencephalographic Abnormalities and Its Associated Factors among Children with Autism Spectrum Disorder in Thailand

**DOI:** 10.3390/healthcare10101969

**Published:** 2022-10-08

**Authors:** Duangkamol Tangviriyapaiboon, Patrinee Traisathit, Vorasith Siripornpanich, Chidawan Suyakong, Hataichanok Apikomonkon, Nontiya Homkham, Salinee Thumronglaohapun, Pimwarat Srikummoon

**Affiliations:** 1 Rajanagarindra Institute of Child Development, Chiang Mai 50180, Thailand; 2 Department of Statistics, Faculty of Science, Chiang Mai University, Chiang Mai 50200, Thailand; 3 Data Science Research Center, Department of Statistics, Faculty of Science, Chiang Mai University, Chiang Mai 50200, Thailand; 4 Research Center in Bioresources for Agriculture, Industry and Medicine, Department of Statistics, Faculty of Science, Chiang Mai University, Chiang Mai 50200, Thailand; 5 Research Center for Neuroscience, Institute of Molecular Biosciences, Mahidol University, Nakhonpathom 73170, Thailand; 6 Department of Occupational Therapy, Faculty of Associated Medical Sciences, Chiang Mai University, Chiang Mai 50200, Thailand; 7 Faculty of Public Health, Thammasat University, Pathumthani 12120, Thailand

**Keywords:** autism spectrum disorder, electroencephalography, EEG abnormalities, epilepsy, Thai autism treatment evaluation checklist, short sensory profile

## Abstract

Epilepsy often causes more severe behavioral problems in children with autism spectrum disorder (ASD) and is strongly associated with poor cognitive functioning. Interestingly, individuals with ASD without a history of epilepsy can have abnormal electroencephalographic (EEG) activity. The aim of this study was to examine associations between EEG abnormalities and the ASD severity in children. The children with ASD who enrolled at the Rajanagarindra Institute of Child Development, Thailand were included in this study. The severity of ASD was measured by interviewing their parents with the Thai autism treatment evaluation checklist. The short sensory profile checklist was used for screening the abnormality of children in each domain. Ordinal logistic regression analysis was used to examine associations between factors potentially linked to EEG abnormalities. Most of the study participants were boys (87.5%) and the median age was 5 years. Among the 128 children, 69.5% showed EEG abnormalities (41.4% slow-wave and 28.1% epileptiform-discharge). The results show that a larger number of symptoms and increased severity of ASD were independently associated with a higher risk of EEG abnormalities. Our results emphasize the need for guidelines on the presence of EEG abnormalities in children with ASD for the early detection of epilepsy and improving treatment outcomes.

## 1. Introduction

Autism spectrum disorder (ASD) is a neurodevelopmental condition in which the phenotype encompasses various degrees of disability, deficits in social communication and interaction, and an unusually restricted and repetitive repertoire of behaviors and interests [1]. In recent decades, ASD has become a major public health problem due to a dramatic increase in its prevalence worldwide to approximately 1 in 160 children [2]. The outcomes of studies carried out during the last two decades indicate that North America and Europe have the highest prevalence of ASD with median rates of 0.90% and 0.61%, respectively. Although the previously reported incidence rate of ASD in children aged less than 12 years old in Thailand was 1.43 per 10,000 in 1998 and 6.94 per 10,000 in 2002 [3,4], the most recently reported prevalence in children aged less than 5 years old is approximately 1 in 161 [5], and the number of new cases is likely to continue increasing. Early detection of ASD facilitates early intervention, which can help in reducing lifetime health care costs substantially. It is estimated that the lifetime cost of autism can be as high as USD 2 million per person, and the broadening of diagnostic criteria with successive versions of the diagnostic and statistical manual of mental disorders (DSM), are likely contributors to changes in prevalence estimates [6,7].

ASD encompasses several other types of problems, such as difficulty sleeping and behavioral issues, such as aggression, impulsivity, tantrums, and attention deficit [8]. Epilepsy seems to be a major contributing factor to the severity of behavioral problems in children with ASD and is strongly associated with poor cognitive functioning [9]. Meanwhile, only 8% of children with ASD are diagnosed with intellectual disability in the absence of epilepsy [10], while the prevalence of epilepsy in children with ASD is estimated to be 5–46% compared to 2–3% in the general population [11,12]. Electroencephalography (EEG), a widely used technique to examine brain activity during rest or evoked brain responses, is a tool that can be used to detect epilepsy. There are two primary methods for examining resting EEG recordings: (1) Visual inspection of the recorded output performed by an EEG expert (standard-EGG), and (2) the computer-analyzed reading of the EEG (C-EEG). The first is capable of detecting significant generalized or focal slowing of the wakeful frequencies as well as detecting any form of paroxysmal or epileptiform activity, whereas the second provides the ability to assess the topography of any detected change (mapping), assess the deeper cerebral sources of detected abnormalities (source localization), and examine the linearity and non-linearity of the recorded signal (complexity analysis) [13]. Interestingly, individuals with ASD without a history of epilepsy can have abnormal EEG activity, including epileptiform-discharge [8]. The reported prevalence of epileptiform-discharge among ASD children has varied from 6.0–59.4% in previous studies [8,14,15,16,17,18,19,20,21,22]. The frequency of epilepsy in the ASD patients was in the range of 5–46% [23].

Despite the high comorbidity of epileptic activity and ASD, how the presence of EEG abnormalities contributes to the symptom presentation of ASD remains unclear [8,13]. The aim of the present study is to evaluate the short sensory profile and the association between EEG abnormalities and ASD among children in Thailand toward the early detection, service, and treatment of ASD in the affected children.

## 2. Materials and Methods

### 2.1. Participants

A cross-sectional observational study was conducted from October 2016 to June 2018. The data were drawn from children with ASD who enrolled at the Rajanagarindra Institute of Child Development, Thailand, and were 3–8 years old on the date of data collection. The required sample size was calculated based on the approach by [24] to find the minimum number of participants required for the study when the size of the study population is unknown. Therefore, the sample size was calculated based on the prevalence of both ASD and epilepsy as follows:

(1) $$n=\left[Z_{\alpha /2}^{2}\cdot P(1-P)\right]{/e}^{2}\;=\left[\left(1.96^{2}\right)\left(0.46\right)\left(0.54\right)\right]/\left(0.10^{2}\right)=95.43\approx 96,$$

where *n* is the required sample size, α is the level of significance (0.05 in this study, and thus *Z*^2^_0.05/2_ = 1.96), *e* is the acceptable error level (10% in this study), and *P* is the prevalence of both ASD and epilepsy (assumed to be 46% based on the highest reported prevalence of epilepsy with ASD [20]. Therefore, at least 127 participants were required for this study. Finally, of the 128 children with ASD, 14 (11%) had a history of epilepsy. The classification of epilepsy was in accordance with ILAE 2017 [25,26].

### 2.2. Inclusion and Exclusion Criteria

All of the participants had already been diagnosed with ASD according to the criteria in the fifth edition of the DSM (DSM-5) [1] by a doctor who is specialized in autism. Additionally, each participant presented with at least one of the following symptoms: (1) Developmental regression/global developmental delay (GDD), (2) stereotypic behavior, (3) inflexible behavior, (4) sensory modulation disorder, (5) aggression, and (6) impulsivity. Exclusion criteria included (1) intractable or tumor-related epilepsy, (2) visual impairment, (3) hearing impairment, (4) multiple disabilities or (5) taking benzodiazepines.

### 2.3. Procedure and Data Collection

The demographic characteristics of the participants comprising gender, age, short sensory profile (SSP) results, and EEG test results were collected. The SSP checklist [27] is a shortened form the Thai version of the sensory profile in screening sensory processing ability of Thai children [28]. The SSP checklist comprised 7 items, including the tactile, taste, movement, seek, auditory, sensory low, and visual levels and scores ranged from 38–190 points. The score was defined as ‘normal’ (38–141 points), ‘abnormal’ (142–154 points) or ‘clearly abnormal’ (155–190 points).

The parents of the children with ASD were interviewed by a multidisciplinary medical team to assess the ASD severity using the Thai Autism Treatment Evaluation Checklist (Thai-ATEC) assessment tool [29] that includes 4 subscales: (1) Speech/language/communication (14 items); (2) sociability (20 items); (3) sensory/cognitive awareness (18 items); and (4) health/physical/behavior (25 items). The Thai-ATEC total scores ranged from 0–179 with maximum scores on the subscales of 28 (speech/language/communication), 40 (sociability), 36 (sensory/cognitive awareness), and 75 (health/physical/behavior). The Thai-ATEC was selected since it has a clear scoring system for distinguishing the severity of the condition (i.e., there is a cut point for each severity level), which the DSM-5 does not have. The overall Thai-ATEC level was defined as mild (1–38 points), moderate (39–67 points) or severe (≥68 points).

After selection of the study participants, the parents of the children were consulted by the clinical team overseeing the EEG recordings and were provided with the opportunity of viewing the exam room, the décor and light of which had been designed to be soothing. During this visit, the parents were informed about how to prepare for the EEG recording, including waking the child earlier than usual on the day of the examination and avoiding ingesting any products containing caffeine to maximize the possibility of the child sleeping during the study. If the participants were unable to sleep, a doctor or pharmacist sedated them with chloral hydrate (20–50 mg/kg with a maximum dose of 2 g) [30]. A doctor, nurse or pharmacist monitored the participants throughout the EEG process. The EEG protocol used in the current study is similar to the standard EEG recording protocol for children [31]. The Ag/AgCl EEG electrodes were applied to the participant’s head by an experienced EEG technologist, after which the EEG sleep pattern was recorded for 30 min using a NicoletOne vEEG32-channel EEG system with an impedance of <10 Ω. The EEG electrodes were performed utilizing the standard international 10–20 system, described as follows: Fp1, Fp2, F3, F4, F8, F7, Fz, A1, T3, C3, Cz, C4, A2, T5, P3, P4, Pz, T6, T4, O1, O2, REFx2, and GND located in the 5 areas: (1) Frontal, (2) temporal, (3) parietal, (4) occipital, and (5) central/mid. All the EEGs were interpreted visually and separately by three pediatric neurologists who did not know about the patient’s underlying illness and the severity of their ASD. Slowing activity is defined as excessive delta and theta activity at various stages of sleep, in relation to the presence or absence of sleep architecture. If the slowing activity was consistently presented and did not change when the patient’s stage of alertness changed at the specific areas, it was reported as abnormal. The slowing activity will be reported when the results from 2/3 of the readers are the same. The EEG results were defined as normal, abnormal in the left, abnormal in the right, and abnormal in the left and right. The overall EEG results were classified into 3 groups: Normal, slow-wave, and epileptiform-discharge. Participants exhibiting electroencephalograms with both slow-wave and epileptiform-discharge components were classed as having composite EEG abnormalities. The study information was revalidated before being compiled and used for statistical analysis.

### 2.4. Ethical Approval and Consent to Participate

Ethical approval for this study was obtained from the Rajanagarindra Institute of Child Development ethics committee (No. 1/2018). The parents gave consent after having been informed about the aim of the study.

### 2.5. Statistical Analysis

The participants’ demographic characteristics, as well as their SSP, Thai-ATEC, and EEG results, are presented as medians and interquartile ranges (IQRs) for continuous variables and frequencies and percentages for categorical variables. The Fisher’s exact test was used to examine the association between symptom diagnosis and EEG results, Thai-ATEC and EEG results, and SSP and EEG results.

Ordinal logistic regression analysis was used to examine the association between factors potentially associated with EEG abnormalities. Factors with a *p*-value < 0.200 in the univariable analysis were included in the multiple ordered logistic regression analysis via backward elimination [32]. All data analyses were performed using Stata version 15 [33].

## 3. Results

The 128 children with ASD who participated in this cross-sectional study had a median age of 4.8 years old (IQR: 3.9–6.2) and the majority were boys (87.5%). Fourteen children (11%) had a history of epilepsy (focal type 9.4% and generalized type 1.6%). Fifty-three children (41.4%) had a slow-wave EEG result, whereas 36 children (28.1%) had an epileptiform-discharge EEG result. EEG abnormalities were found in 65 (50.8%) on both sides of the frontal part, 12 (9.4%) were detected equally on both sides of the occipital part and the central or mid part, and 5 (3.9%) were detected on both sides of the temporal part, while 121 (94.5%) had a normal electroencephalogram of the parietal part. From the total SSP results, 57% were identified as clearly abnormal and abnormal, among whom 71.1%, 56.3%, and 50% were in the seek, visual, and auditory levels, respectively. From the Thai-ATEC results, 53.9% and 37.5% were classified as moderate and severe, respectively (Table 1).

The majority of the children with ASD presented with three symptoms (51/128; 39.8%), followed by two symptoms (29/128; 22.7%), and four symptoms (23/128; 18.0%), whereas only one child (0.8%) presented with six symptoms. Moreover, 25.49% (13/51) of the children presented with three symptoms (stereotypic, aggression, and impulsive behavior), followed by stereotypic sensory modulation disorder and impulsive behavior in 23.53% (12/51). Furthermore, 62.1% (18/29) of the children presented with two symptoms (stereotypic and impulsive behavior) and 34.8% (8/23) of the ASD children presented with four symptoms, developmental regression/GDD, stereotypic, aggression, and impulsive behavior or stereotypic, sensory modulation disorder, aggression, and impulsive behavior (Table 2).

**Figure 1:**
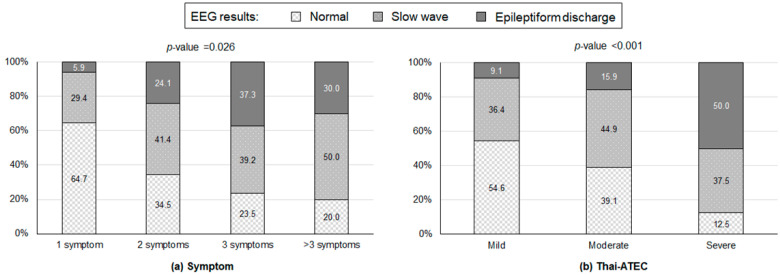
Electroencephalography (EEG) results according to (**a**) the number of diagnosed autism spectrum disorder (ASD) symptoms and (**b**) Thai-ATEC (Thai autism treatment evaluation checklist) results. *p*-Values were obtained from Fisher’s exact tests.

ASD children with one symptom were more likely to have a normal electroencephalogram than those with two, three, and more than three symptoms (64.7% vs. 34.5%, 23.5%, and 20.0%, respectively), while those with three or more than three symptoms were more likely to have an epileptiform-discharge EEG result than those with one and two symptoms (37.3%, 30.0% vs. 5.9%, 24.1%; *p*-value = 0.026) (Figure 1a). The prevalence of epileptiform-discharge tended to be higher along with the severity of ASD symptoms evaluated using Thai-ATEC (50.0%, 15.9%, and 9.1% for severe, moderate, and mild symptoms, respectively (*p*-value < 0.001) (Figure 1b).

**Figure 2:**
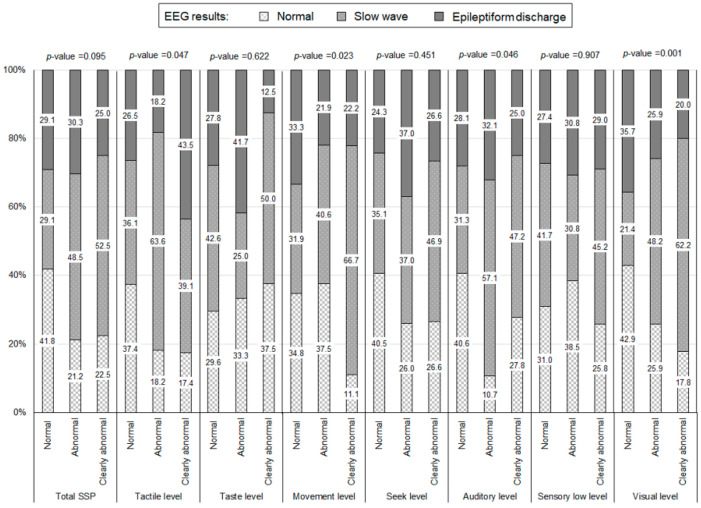
Electroencephalography (EEG) results according to the short sensory profile (SSP). *p*-Values were obtained from Fisher’s exact test.

In addition, children with a tactile level SSP result that was clearly abnormal were more likely to have an epileptiform-discharge EEG result than those with normal or abnormal results (43.5% vs. 26.5% and 18.2%, respectively; *p*-value = 0.047). Of the children with a movement level SSP result, the clearly abnormal group was more likely to have a slow-wave EEG result than those in the normal and abnormal groups (66.7% vs. 31.9% and 40.6%, respectively; *p*-value = 0.023). Of the children with a visual level SSP result, the clearly abnormal group was more likely to have a slow-wave EEG result than those in the normal and abnormal groups (62.2% vs. 21.4% and 48.2%, respectively; *p*-value = 0.001) (Figure 2).

According to the multivariable ordinal logistic regression analysis, factors associated with an abnormal EEG result were ASD children who presented with more than two symptoms (adjusted odds ratio [aOR] = 5.14; 95% confidence interval [95% CI] = 1.66–15.96), two symptoms (aOR = 3.74; 95% CI = 1.07–13.02) or having a severe Thai-ATEC level (aOR = 6.25; 95% CI = 1.62–24.20) (Table 3). The positive predictive value (PPV) of diagnosed symptom and Thai-ATEC score-predicted abnormal EEG result was 74.1%, while the negative predictive value (NPV) of diagnosed symptom and Thai-ATEC score-predicted normal EEG result was 62.5%. The sensitivity and the specificity of this model were 93.3% and 25.6%, respectively (Table 4).

- Sensitivity: 93.3% (95% confidence interval [95% CI]: 85.9–97.5)
- Specificity: 25.6% (95% CI: 13.0–42.1)
- Receiver operating characteristic (ROC) area: 0.59 (95% CI: 0.52–0.67)
- Positive predictive value (PPV): 74.1% (95% CI: 65.0–81.9)
- Negative predictive value (NPV): 62.5% (95% CI: 35.4–84.8)

The probability of an abnormal EEG result according to the number of symptoms and Thai-ATEC levels by applying the predictive model is presented in Table 5. Herein, 44.85% and 47.60% of ASD children who presented with two symptoms and had a severe or moderate Thai-ATEC level, respectively, had a slow-wave EEG result. In addition, 45.31% and 53.29% of ASD children who presented with more than two symptoms and had moderate or severe Thai-ATEC level, respectively, had an epileptiform-discharge EEG result.

## 4. Discussion

A prospective study involving 128 children diagnosed with ASD to evaluate the association between the results of ASD, EEG, and SSP was conducted at Rajanagarindra Institute of Child Development in Chiang Mai, Thailand. Most of the study participants were male (87.5%) aged 5 years old (IQR = 4–7 years old) with some (11%) having a history of epilepsy. Among the 128 children, 69.5% showed EEG abnormalities (41.4% slow-wave and 28.1% epileptiform-discharge). Although the abnormal EGG rate in our study was similar to those reported in previous studies (66–75%) [8,12]. However, the rate of epileptiform-discharge EEG results presented in our study was lower (28.1% vs. 59.4%). Mulligan and Trauner (2014) [8] suspected that the high prevalence in their studies might have been related to prolonged EEG recordings that captured both wakefulness and a full night’s sleep and suggested a longer recording time, which gives a truer assessment of epileptiform-discharge. Therefore, we will certainly consider recording electroencephalograms over a longer period to more precisely evaluate the prevalence of EEG abnormalities in future studies.

We found that ASD children with two or more than two symptoms had a 3-fold and 5-fold higher risk of an abnormal EEG result compared to those with only one symptom. This emphasized the fact that the higher the number of ASD symptoms, the higher the risk of EEG abnormalities. To the best of our knowledge, this association between the number of symptoms and EEG abnormalities has not previously been highlighted. However, the authors of a retrospective study that considers the risk of specific ASD symptoms found that an increased severity of the symptom resulted in a higher risk of EEG abnormalities [8]. They also found that children with stereotypic and aggressive behavior were more likely to have epileptiform activity.

Children with a severe Thai-ATEC level also had a 6-fold higher risk of EEG abnormalities compared to those with mild or moderate Thai-ATEC levels. The findings from a previous study in India suggest that children with severe ASD commonly present with epilepsy [22]. Although they found a similar trend of association between ASD severity and epileptiform-discharge, it was not statistically significant.

According to the regression analysis, there was no association between the SSP and the EEG abnormalities. However, we found that children identified with clearly abnormal tactile, movement, and visual domains seemed more likely to have a slow-wave EEG result than those with normal or abnormal levels. In addition, we also provided a model to predict the probability of EEG abnormalities in nine patterns based on the symptoms and severity of ASD. This model yielded high sensitivity (93.3%) concerning the risk of EEG abnormalities. Although it yielded a low specificity, its positive and negative predictive values were acceptable (74.1% vs. 62.5%). Predicting the EEG result using this model could be helpful in the early detection and intervention of ASD in children identified as at risk. However, further study with additional potential associated factors might improve the sensitivity and specificity of the predictive model.

This study had some limitations. First, we did not examine the possible association between specific ASD symptoms and EEG abnormalities due to the fact that the number of children with some of the ASD symptoms was insufficient for performing an ordinal logistic regression analysis. Second, some factors associated with EEG abnormalities, such as formal language testing and IQ testing were not included in this study. Third, we did not examine the possible association between a history of epilepsy and ASD severity due to the number of children with a history of epilepsy, which offers an insufficient sample size for performing the correlation analysis. However, we adjusted for several potential factors associated with EEG abnormalities in the analysis, including the number of symptoms using the Thai-ATEC evaluation system with the severity of ASD and the SSP results.

## 5. Conclusions

In this study, we revealed an association between a higher number of symptoms and the severity of ASD with an increased risk of EEG abnormalities (i.e., slow-wave and epileptiform-discharge). Currently, although there are clinical practice guidelines for diagnosing epilepsy in Thailand, identifying EEG abnormalities that suggest epilepsy in young children with ASD is not yet part of the process [34]. Our results emphasize the need for guidelines on the presence of EEG abnormalities in children with ASD, with referrals to perform the EEG for the proper management of EEG abnormality, and referrals to pediatric neurologists for diagnosing epilepsy with ASD early on, thereby improving treatment outcomes.

## Figures and Tables

**Table 1:** Participant characteristics and electroencephalography (EEG), SSP (short sensory profile), and Thai-ATEC (Thai autism treatment evaluation checklist) results (*n* = 128).

Variable			Number	Percent
Characteristic				
	Gender			
		Female	16	12.5
		Male	112	87.5
	Age (years old)			
		Median (Interquartile range)	4.8 (3.9–6.2)	
	History of epilepsy			
		Focal type	12	9.4
		Generalized type	2	1.6
		No	114	89.0
EEG testing				
	Overall EEG results			
		Normal	39	30.5
		Slow-wave	53	41.4
		Epileptiform-discharge	36	28.1
	EEG of the frontal part			
		Normal	48	37.5
		Abnormal on the left	9	7.0
		Abnormal on the right	6	4.7
		Abnormal on the left and right	65	50.8
	EEG of the temporal part			
		Normal	120	93.7
		Abnormal on the left	1	0.8
		Abnormal on the right	2	1.6
		Abnormal on the left and right	5	3.9
	EEG of the parietal part			
		Normal	121	94.5
		Abnormal on the left	3	2.4
		Abnormal on the right	1	0.8
		Abnormal on the left and right	3	2.3
	EEG of the occipital part			
		Normal	114	89.0
		Abnormal on the left	2	1.6
		Abnormal on the left and right	12	9.4
	EEG result in on central/mid part			
		Normal	116	90.6
		Abnormal	12	9.4
SSP				
	Tactile level			
		Clearly abnormal	23	18.0
		Abnormal	22	17.2
		Normal	83	64.8
	Taste level			
		Clearly abnormal	8	6.2
		Abnormal	12	9.4
		Normal	108	84.4
	Movement level			
		Clearly abnormal	27	21.1
		Abnormal	32	25.0
		Normal	69	53.9
	Seeking level			
		Clearly abnormal	64	50.0
		Abnormal	27	21.1
		Normal	37	28.9
	Auditory level			
		Clearly abnormal	36	28.1
		Abnormal	28	21.9
		Normal	64	50.0
	Sensory low level			
		Clearly abnormal	31	24.2
		Abnormal	13	10.2
		Normal	84	65.6
	Visual level			
		Clearly abnormal	45	35.2
		Abnormal	27	21.1
		Normal	56	43.7
	Overall			
		Clearly abnormal	40	31.2
		Abnormal	33	25.8
		Normal	55	43.0
Thai-ATEC				
	Overall Thai-ATEC level			
		Mild	11	8.6
		Moderate	69	53.9
		Severe	48	37.5

Abbreviations: EEG, electroencephalography; Thai-ATEC, Thai autism treatment evaluation checklist; SSP, short sensory profile.

**Table 2:** ASD children presented with symptoms (n = 128).

Symptoms	n	N	Percent
One diagnosed			
- Developmental regression/GDD	1	17	5.9
- Stereotypic	2	17	11.8
- Inflexible behavior	2	17	11.8
- Aggression	1	17	5.9
- Impulsive	11	17	64.7
Two diagnosed			
- Developmental regression/GDD + Stereotypic	3	29	10.3
- Stereotypic + Sensory modulation disorder	3	29	10.3
- Stereotypic + Aggression	1	29	3.5
- Stereotypic + Impulsive	18	29	62.1
- Inflexible behavior + Impulsive	2	29	6.9
- Aggression + Impulsive	2	29	6.9
Three diagnosed			
- Developmental regression/GDD + Stereotypic + Inflexible behavior	1	51	1.96
- Developmental regression/GDD + Stereotypic + Aggression	1	51	1.96
- Developmental regression/GDD + Stereotypic + Impulsive	5	51	9.8
- Developmental regression/GDD + Inflexible behavior + Impulsive	1	51	1.96
- Developmental regression/GDD + Sensory modulation disorder + Aggression	1	51	1.96
- Developmental regression/GDD + Aggression + Impulsive	4	51	7.84
- Stereotypic + Inflexible behavior + Sensory modulation disorder	1	51	1.96
- Stereotypic + Inflexible behavior + Impulsive	3	51	5.88
- Stereotypic + Sensory modulation disorder + Aggression	4	51	7.84
- Stereotypic + Sensory modulation disorder + Impulsive	12	51	23.53
- Stereotypic + Aggression + Impulsive	13	51	25.49
- Inflexible behavior + Sensory modulation disorder + Impulsive	2	51	3.92
- Inflexible behavior + Aggression + Impulsive	1	51	1.96
- Sensory modulation disorder + Aggression + Impulsive	2	51	3.92
Four diagnosed			
- Developmental regression/GDD + Stereotypic + Inflexible behavior + Aggression	1	23	4.4
- Developmental regression/GDD + Stereotypic + Sensory modulation disorder + Impulsive	2	23	8.7
- Developmental regression/GDD + Stereotypic + Aggression + Impulsive	8	23	34.8
- Stereotypic + Inflexible behavior + Sensory modulation disorder + Impulsive	3	23	13.0
- Stereotypic + Inflexible behavior + Aggression + Impulsive	1	23	4.4
- Stereotypic + Sensory modulation disorder + Aggression + Impulsive	8	23	34.8
Five diagnosed			
- Developmental regression/GDD + Stereotypic + Inflexible behavior + Sensory modulation disorder + Impulsive	1	6	16.7
- Developmental regression/GDD + Stereotypic + Inflexible behavior + Aggression + Impulsive	1	6	16.7
- Developmental regression/GDD + Stereotypic + Sensory modulation disorder + Aggression + Impulsive	3	6	50.0
- Stereotypic + Inflexible behavior + Sensory modulation disorder + Aggression + Impulsive	1	6	16.7
- Six diagnosed			
- Developmental regression/GDD + Stereotypic + Inflexible behavior + Sensory modulation disorder + Aggression + Impulsive	1	1	100.0

Abbreviations: GDD, global developmental delay.

**Table 3:** The predictive model for factors associated with an abnormal electroencephalograph.

Variable		Univariable Ordinal Logistic Regression					Multivariable Ordinal Logistic Regression				
		cOR	95% CI of cOR			*p*-Value	aOR	95% CI of aOR			*p*-Value
Gender						0.564					
	Female (ref.)	1.00									
	Male	1.33	0.50	-	3.56		-	-	-	-	-
History of epilepsy						0.513					
	No (ref.)	1.00									
	Yes	0.70	0.24	-	2.04		-	-	-	-	-
Tactile level						0.118					
	Normal (ref.)	1.00					-	-	-	-	-
	Abnormal	1.32	0.58	-	3.02		-	-	-	-	-
	Clearly abnormal	2.52	1.05	-	6.05		-	-	-	-	-
Taste level						0.638					
	Normal (ref.)	1.00					-	-	-	-	-
	Abnormal	1.31	0.41	-	4.22		-	-	-	-	-
	Clearly abnormal	0.59	0.16	-	2.17		-	-	-	-	-
Movement level						0.423					
	Normal (ref.)	1.00					-	-	-	-	-
	Abnormal	0.71	0.32	-	1.56		-	-	-	-	-
	Clearly abnormal	1.31	0.59	-	2.87		-	-	-	-	-
Seeking level						0.334					
	Normal (ref.)	1.00					-	-	-	-	-
	Abnormal	2.00	0.78	-	5.12		-	-	-	-	-
	Clearly abnormal	1.51	0.71	-	3.22		-	-	-	-	-
Auditory level						0.156					
	Normal (ref.)	1.00					-	-	-	-	-
	Abnormal	2.23	0.99	-	5.02		-	-	-	-	-
	Clearly abnormal	1.30	0.61	-	2.78		-	-	-	-	-
Sensory low level						0.879					
	Normal (ref.)	1.00					-	-	-	-	-
	Abnormal	0.89	0.29	-	2.72		-	-	-	-	-
	Clearly abnormal	1.18	0.55	-	2.50		-	-	-	-	-
Visual level						0.786					
	Normal (ref.)	1.00					-	-	-	-	-
	Abnormal	1.22	0.52	-	2.89		-	-	-	-	-
	Clearly abnormal	1.28	0.62	-	2.65		-	-	-	-	-
Symptoms						0.002					0.018
	1 (ref.)	1.00									
	2	3.72	1.12	-	12.31		3.74	1.07	-	13.02	
	>2	6.56	2.23	-	19.30		5.14	1.66	-	15.96	
Thai-ATEC						<0.001					<0.001
	Mild (ref.)	1.00					1.00				
	Moderate	1.86	0.54	-	6.37		1.36	0.38	-	4.89	
	Severe	9.23	2.48	-	34.34		6.25	1.62	-	24.20	

Abbreviations: Thai-ATEC, Thai Autism Treatment Evaluation Checklist; ref., reference group; cOR, crude odds ratio; aOR, adjusted odds ratio; 95% CI, 95% confidence interval.

**Table 4:** Electroencephalography (EEG) classification with the predictive model.

Predictive Model Results	Actual EEG Results					
	Normal (n = 39)		Slow-Wave (n = 53)		Epileptiform-Discharge (n = 36)	
	n/N	%	n/N	%	n/N	%
Normal (n = 16)	10/16	62.5	6/16	37.5	0/16	0.0
Slow-wave (n = 67)	25/67	37.3	29/67	43.3	13/67	19.4
Epileptiform-discharge (n = 45)	4/45	8.9	18/45	40	23/45	51.1

**Table 5:** The probability of an abnormal electroencephalograph from model prediction according to the number of symptoms and the Thai-ATEC (Thai autism treatment evaluation checklist) level.

			Probability of an Abnormal EEG (Percent)		
Pattern	Symptoms	Thai-ATEC Level	Normal	Slow-Wave	Epileptiform-Discharge
1	1	Mild	77.13	19.44	3.43
2	1	Moderate	47.44	40.86	11.70
3	1	Severe	39.60	44.97	15.43
4	2	Mild	71.23	24.16	4.61
5	2	Moderate	39.86	44.85	15.29
6	2	Severe	32.50	47.60	19.90
7	>2	Mild	35.03	46.82	18.15
8	>2	Moderate	12.61	42.08	45.31
9	>2	Severe	9.49	37.22	53.29

## Data Availability

The datasets used and/or analyzed during the current study are available from the corresponding author on reasonable request.
